# A Rare Cause of Severe Bioprosthetic Transvalvular Regurgitation Diagnosed by Transesophageal Echocardiogram

**DOI:** 10.1016/j.case.2023.11.005

**Published:** 2023-12-23

**Authors:** Aleef Mannan, Marie Caruso, Enrique Pantin

**Affiliations:** Department of Anesthesiology and Perioperative Medicine, Robert Wood Johnson University Hospital, Rutgers Robert Wood Johnson Medical School, New Brunswick, New Jersey

**Keywords:** Bioprosthetic mitral valve, Mitral regurgitation, Suture snare, Restricted leaflet, Transesophageal echocardiogram

## Abstract

• Suture snare of a strut may cause severe transvalvular MR after bioprosthetic MVR.
• Intraoperative TEE is vital in evaluating hemodynamic instability in MVR surgery.
• 3D imaging improves the diagnosis of bioprosthetic dysfunction post-MVR.

## Graphical abstract

**Figure:**
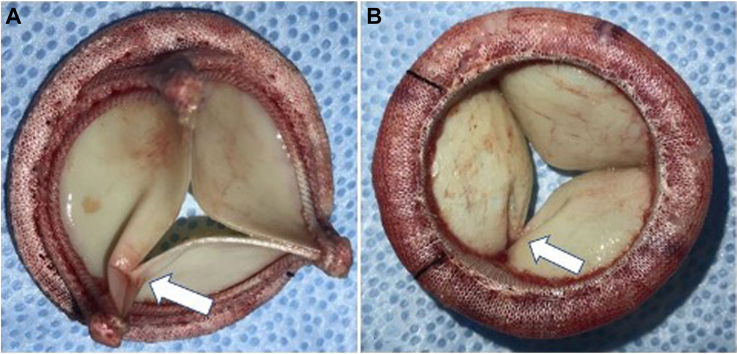

## Introduction

Mitral valve (MV) replacement (MVR) with a bioprosthetic valve is a widely used treatment for mitral regurgitation (MR). Complications with bioprosthetic MVR immediately postimplantation are rare and include paravalvular leak, stuck leaflets, and valve malposition and damage, among other considerations.^1^ We present a patient with severe transvalvular regurgitation after bioprosthetic MVR diagnosed with transesophageal echocardiogram (TEE) due to unintended suture encircling of a replacement valve strut.

## Case Presentation

A 64-year-old woman with a medical history of hypertension, diabetes mellitus type 2, and MV prolapse with severe MR underwent a MV repair with annuloplasty via right minithoracotomy approach 1 month ago. The patient presented with new-onset atrial fibrillation and progressively worsening dyspnea on exertion and was found to have recurrent severe MR post–MV repair. They returned for right minithoracotomy MVR and left atrial appendage ligation.

**Figure 1:**
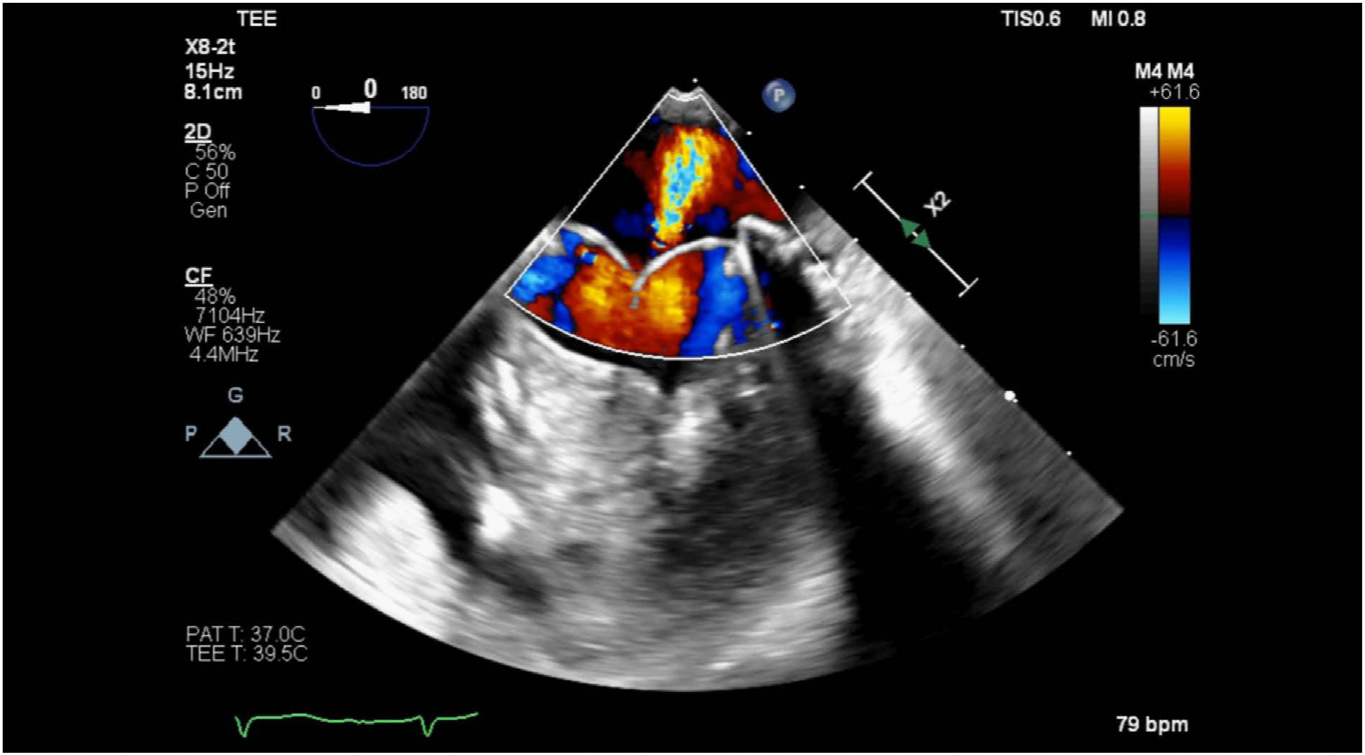
Two-dimensional TEE, midesophageal 4-chamber view (foreshortened) with color-flow Doppler, systolic phase, demonstrates MR.

**Figure 2:**
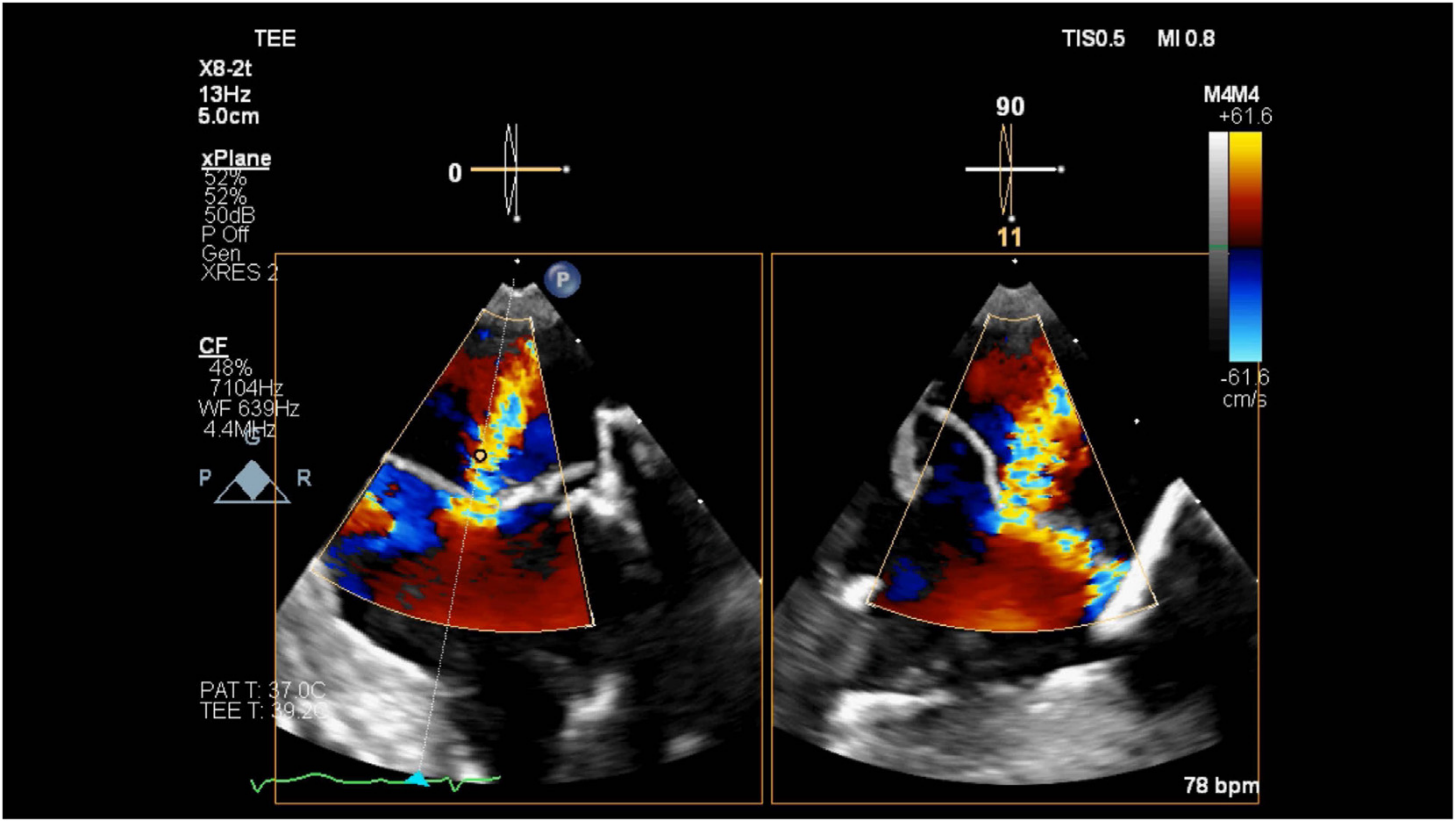
Two-dimensional TEE, midesophageal 4- (*left*) and 2- (*right*) chamber views (foreshortened) with color-flow Doppler, systolic phase, demonstrates severe valvular MR following bioprosthetic MVR.

**Figure 3:**
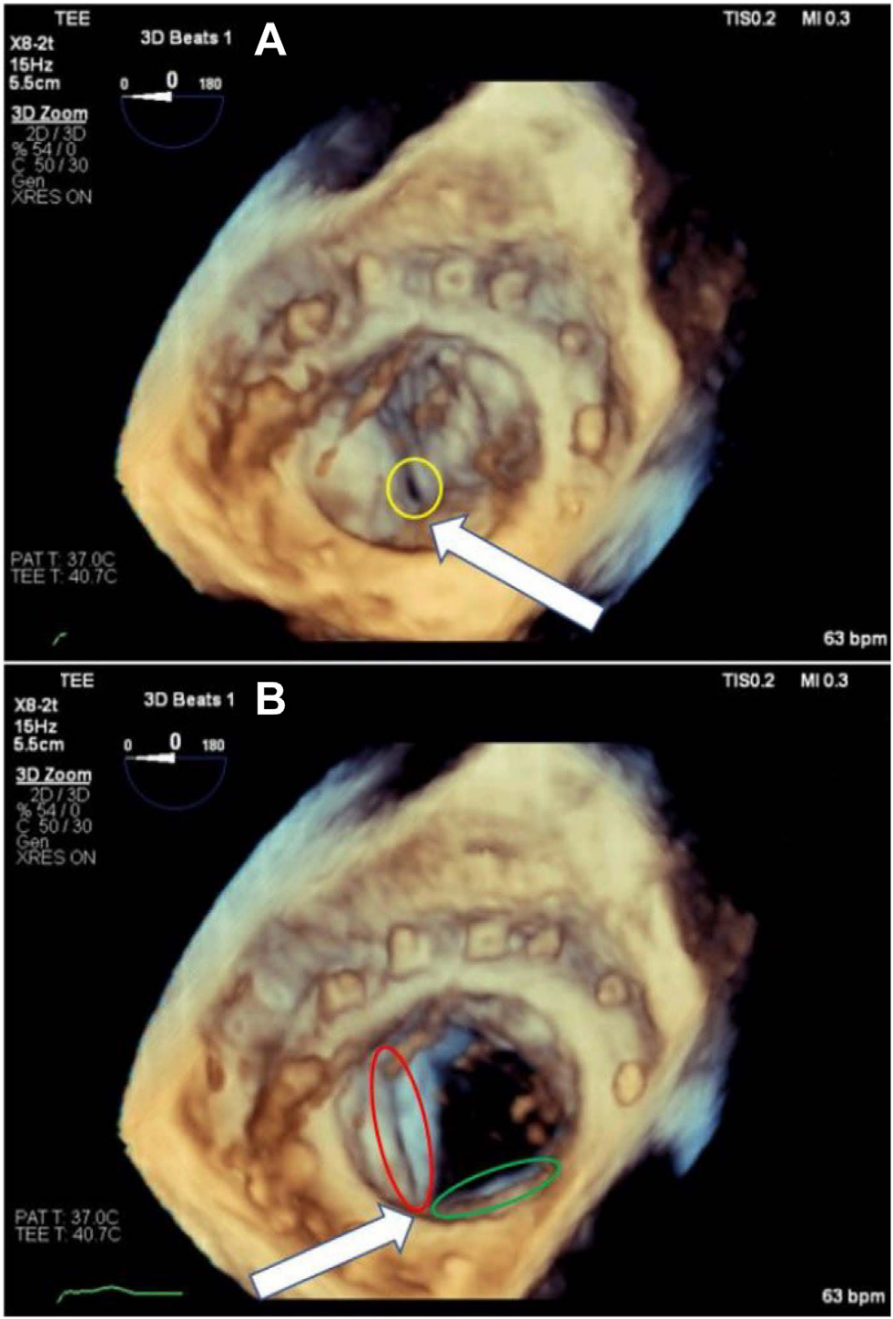
Three-dimensional TEE, volume-rendered short-axis en face display (surgeon's view) of the MVR from the atrial perspective in systole **(A)** and diastole **(B)**, demonstrates the disrupted posterior commissure with lack of leaflet coaptation (*yellow circle*) and folding of the anterolateral (*red circle*) and posteromedial (*green circle*) leaflets resulting in restricted leaflet motion.

The patient underwent an uneventful general endotracheal anesthetic. Initial intraoperative TEE revealed severe MR and normal left ventricle function. Due to scar tissue and lung adhesion to the chest wall, the right minithoracotomy approach was abandoned and converted to open sternotomy. The mitral ring was successfully removed, and the MV was replaced with a 29 mm bovine pericardial tissue valve sutured in place without hand sutures using the COR-KNOT (Minogue Medical) automatic titanium fastener system. In preparation for separating from cardiopulmonary bypass (CPB), the heart was filled, CPB flow was decreased, and the MV was evaluated by TEE. Moderate MR was noted with the anticipation that this would improve upon complete filling of the left ventricle and separation from CPB (Figure 1, Video 1). The patient was successfully separated from CPB with vasopressor and inotropic support, and the sternotomy was closed. Due to increasing vasopressor requirements prior to skin closure, repeat operative TEE was done to reevaluate the MVR. Transesophageal echocardiogram revealed worsening severe central MR (Figure 2, Video 2). Three-dimensional imaging of the MV showed restricted movement of 2 of the leaflets, causing incomplete coaptation during systole (Figure 3, Video 3).

**Figure 4:**
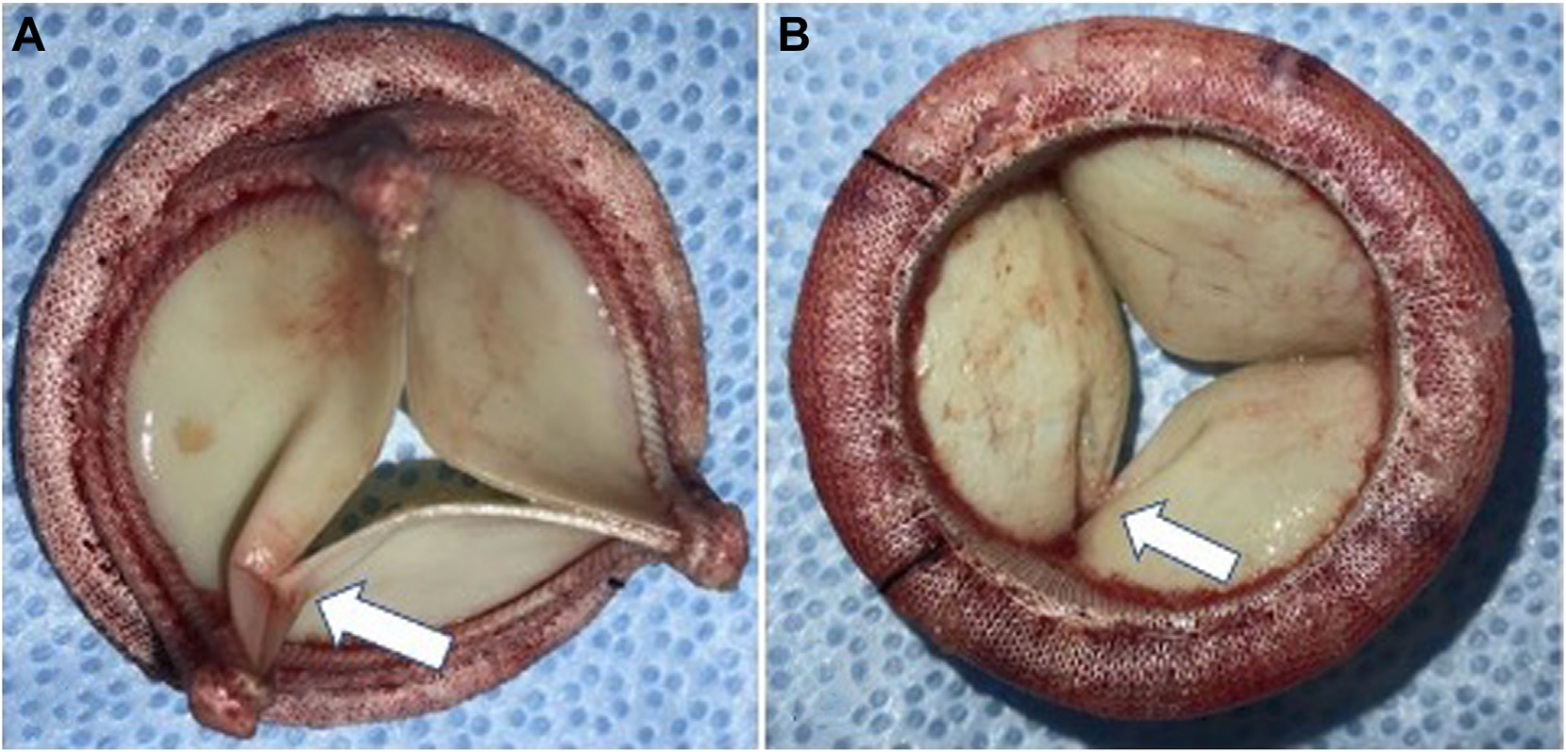
Gross images of the explanted bioprosthetic valve demonstrate the snared strut (*arrows*) on the ventricular **(A)** and atrial **(B)** sides, causing disruption of leaflet commissure and leaflet folding leading to restricted movement.

Although the etiology of the restriction was unclear, it was decided to reopen the chest and replace the dysfunctional valve with a different 29 mm porcine valve without complications. Reevaluation of the new valve showed a well-functioning bioprosthetic MV without any paravalvular or transvalvular leak. The patient tolerated the procedure well, was extubated to nasal cannula at the end of the case, and was transported to the cardiac intensive care unit. The surgeon noted on reopening that a suture loop had snared over one of the valve posts, most likely being the culprit for the significant intravalvular regurgitation, as it tethered 2 of the bioprosthetic leaflets in a manner that restricted its full closure. Postoperative evaluation of the gross specimen revealed where one of the sutures had encircled the replacement valve strut causing disruption of the commissure of 2 of the valve leaflets and its tethering (Figure 4).

## Discussion

This case report describes a rare complication of severe transvalvular regurgitation after bioprosthetic MVR due to suture snare of a strut causing restricted MV leaflet motion at a valve leaflet commissure and incomplete valve leaflet coaptation during systole. The COR-KNOT device used to suture the bioprosthetic valve into place has been shown to reduce CPB time, aortic cross-clamp time, and operative time in addition to improved suture strength (compared to hand-tied sutures).^2^^,^^3^ However, there have been reports of the COR-KNOT being associated with prosthetic valvular regurgitation. In a study of COR-KNOT complications compared with hand-tied sutures, the incidence of transvalvular regurgitation after MVR with the use of COR-KNOT sutures is as high as 4.5%.^4^ In general, the most frequent cause for immobilization of bioprosthetic valve leaflets is due to remnants of the subvalvular apparatus or retained native valve structures interfering with valve closure.^1^^,^^5^ In a case report by Fujiwara *et al.*^6^, paravalvular and transvalvular leak was noted on TEE post–prosthetic valve MVR and was caused by a suture loop jamming around the stent of the bioprosthesis. Takeshita *et al.*^7^ describe a case of a stuck bioprosthetic valve leaflet due to a loop of suture caught on a strut beneath the valve, likely caused by malrotation of the valve holder during placement into the mitral annulus.

It is important to emphasize the role of TEE in the interrogation of a newly placed bioprosthetic heart valve. Immediately post-CPB, intraoperative TEE is an important diagnostic and therapeutic tool in valve replacement surgery and should be widely implemented.^8^ In the case presented above, upon preliminary evaluation of the MVR with two-dimensional TEE, moderate MR was noted (Figure 1). Upon imaging reevaluation, after the case, it appears that the initial images were taken initially of the plane, thus misinterpreting the severity of the regurgitant jet. After completely separating from CPB and sternal closure, due to worsening hemodynamics, the heart and valve were reassessed and worsening MR was noted. Further interrogation of the valve with three-dimensional imaging revealed restriction and abnormal function of 2 leaflets of the valve. In addition, on two-dimensional imaging (Video 1 and Video 2), 1 of the leaflets is abnormally folding in both systole and diastole, which provides imaging evidence for the mechanism of the MR. In this case, TEE was an important diagnostic aid in the search for a source of the patient's worsening hemodynamic stability. This problem might have been identified earlier if a more comprehensive imaging evaluation had been done before sternal closure. Prompt diagnosis of the snared strut allowed for the replacement of the dysfunctional valve in a timely manner, avoiding further hemodynamic compromise.

## Conclusion

Intraoperative two-dimensional and Doppler TEE are invaluable tools in diagnosing and preventing hemodynamic instability prior to separation from CPB in MVR surgeries. This case illustrates the importance of a complete valve assessment immediately post–CPB weaning and before heart decannulation. Three-dimensional imaging of the replaced MV should be utilized to further aid in the prompt diagnosis of bioprosthetic dysfunction immediately post-MVR. Although rare, suture snaring of a strut should remain on the differential when evaluating restricted leaflet motion and transvalvular regurgitation immediately post–bioprosthetic MVR.

## Consent Statement

Complete written informed consent was obtained from the patient (or appropriate parent, guardian, or power of attorney) for the publication of this study and accompanying images.

## Ethics Statement

The authors declare that the work described has been carried out in accordance with The Code of Ethics of the World Medical Association (Declaration of Helsinki) for experiments involving humans.

## Funding Statement

The authors declare that this report did not receive any specific grant from funding agencies in the public, commercial, or not-for-profit sectors.

## Disclosure Statement

The authors report no conflict of interest.

## Supplementary Data

Video 1 Two-dimensional TEE, midesophageal 4-chamber view (foreshortened) with color-flow Doppler, demonstrates MR and the abnormal indentation on the prosthetic leaflet.

Video 2 Two-dimensional TEE, midesophageal 4- (*left*; 0°) and 2- (*right*; 90°) chamber zoom views with color-flow Doppler, demonstrates severe valvular MR following bioprosthetic MVR.

Video 3 Three-dimensional TEE, volume-rendered short-axis en face display (surgeon's view) of the MVR from the atrial perspective, demonstrates the folding of the anterolateral and posteromedial leaflets resulting in restricted leaflet motion.
